# Investigating the demotivation of Moroccan CFL learners from an alienation theory perspective

**DOI:** 10.3389/fpsyg.2025.1471486

**Published:** 2025-06-03

**Authors:** Fei Peng, Qiuyue Lou

**Affiliations:** College of Humanities and Foreign Languages, China Jiliang University, Hangzhou, China

**Keywords:** L2 demotivation, Moroccan CFL learners, alienation theory, mediational factors, formulating mechanism

## Abstract

This study reports on the results of a multi-method investigation on second language (L2) demotivation among Moroccan Learners of Chinese as a Foreign Language (CFL) learners from the perspective of Alienation Theory. A total of 97 Moroccan students with varied majors from a university in East China took part in a questionnaire survey. Then Semi-structured interviews were applied to collect the data. Through Pearson’s correlations, a multiple regression and a multivariate analysis of variance (MANOVA) analysis approach, this study found that L2 demotivation prevailed among the participants, influenced by an array of social isolation or estrangement-mediated and normlessness-mediated factors among classic four alienators. Students at different L2 demotivation levels reacted to alienators differently. Students from the high demotivation group suffered most across all four demotivators, but such gaps appeared most substantially in social isolation or estrangement, followed by meaninglessness, and then powerlessness. No significant difference was found between average demotivators and low demotivators in powerlessness and meaninglessness. Qualitative findings contextualized these results, identifying institutional pressures and sociocultural barriers as key mechanisms driving motivational attrition. The study advances theoretical understanding of L2 demotivation in transnational educational contexts and provides actionable strategies for fostering inclusive CFL pedagogies.

## Introduction

Learning Motivation plays a critical role in successful second language acquisition (Oxford and Shearin, 1994). Not only has positive motivation received a lot of attention, such as enjoyment, resilience and flow (e.g., Jin and Zhang, 2018; Liu and Song, 2021; Liu and Han, 2022), but negative motivation has also been heatedly discussed in recent years (Dewaele et al., 2019), especially after Dörnyei (2001) proposed definition of second language (L2) learning demotivation. After that, more and more researchers have paid close attention to L2 learning demotivation (e.g., Kikuchi, 2009; Karaca and Inan, 2020). To date, most previous studies have focused on describing demotivation and identifying its demotivators in English as a foreign language (EFL) class (Hu and Cai, 2010; Li, 2013; Moiinvaziri and Razmjoo, 2014; Kim et al., 2018). However, few studies have investigated the demotivation of Chinese as a foreign language (CFL) class, which thus necessitates a more comprehensive approach to understand the formulation of L2 demotivation of CFL learners.

Studying abroad constitutes a transitional phase marked by multifaceted physiological, social, and emotional adjustments. Failure to navigate these transitions effectively may lead to maladjustment and psychological challenges, including academic alienation—a phenomenon negatively associated with educational quality and linked to broader institutional issues such as reduced academic achievement and behavioral problems (Newmann, 1981). Universities worldwide face growing complexities in supporting culturally diverse student populations, particularly as international students frequently encounter systemic microaggressions across academic and social environments (Ancis et al., 2000; Solórzano et al., 2000). Compounding these challenges are persistent sociocultural barriers, including social alienation, racial stereotyping, cultural intolerance, and exclusionary practices, which exacerbate psychological distress and hinder integration (Hanassab, 2006; Klomegah, 2006; Lee and Rice, 2007; Sherry et al., 2010). Academic alienation, defined as a “a separation or distance among two or more entities and involves a sense of anguish or loss, resulting in a student viewing life and school as fragmentary and incomplete” (Dean, 1961; Galbo, 1980), correlates strongly with attrition risks, underscoring its significance as a predictor of dropout intentions (Schram and Lauver, 1988; Nottingham et al., 1992).

China’s growing economic influence and expanding global trade partnerships have positioned it as a key destination for international students. In recent years, initiatives such as the Zhejiang-Africa educational cooperation program have further incentivized international enrollment, particularly among Moroccan high school graduates pursuing tertiary education at universities in Hangzhou, Zhejiang Province. Central to these efforts is Chinese as a Foreign Language (CFL) education, which aims to equip students with advanced linguistic proficiency and intercultural communication skills. To achieve this, universities provide daily language courses, including Comprehensive Chinese, Chinese Characters and Writing, and Chinese Listening and Speaking. Proficiency in Chinese is critical for academic success, as it enables students to meet institutional requirements such as passing the HSK (Hanyu Shuiping Kaoshi)—a standardized proficiency test—and obtaining graduation credentials (Liu et al., 2020).

However, emerging observations from educators, including the authors, highlight demotivation as a critical issue among international students in Chinese universities, particularly Moroccan learners. Compared to peers from other countries, Moroccan students exhibited notably higher disengagement in Chinese language courses, manifested through recurrent tardiness, early departures, absenteeism, and midterm attrition. Classroom participation was markedly low: students often avoided discussions, provided minimal responses to questions, and cited non-specific health concerns (e.g., “feeling unwell”) to justify disengagement. Furthermore, social isolation was evident, with many students sitting alone in corners during class and reporting limited interaction with Chinese peers. These behaviors collectively signal a progressive decline in motivation toward Chinese language acquisition. Such patterns underscore the urgency for CFL educators and administrators to critically examine international students’ learning experiences and implement strategies to sustain L2 motivation. This study therefore investigates the contextual and psychosocial factors driving Moroccan students’ demotivation within Chinese higher education settings.

## Literature review

### Research on L2 demotivation

Research on L2 demotivation originated in European contexts (Chambers, 1993; Oxford and Shearin, 1994) and subsequently expanded to Asian EFL settings, including Japan (Kikuchi, 2019; Weda, 2018), South Korea (Kim and Kim, 2015; Song and Kim, 2017), and China (Xie et al., 2018; Zhou, 2012). Early conceptualizations defined demotivation as “external forces that reduce or diminish the motivational basis of a behavioral intention or ongoing action” (Dörnyei, 2001), framing it primarily as an externally driven phenomenon, particularly teacher-related influences. For instance, Chambers (1993) seminal study of 191 British secondary students exclusively ascribed L2 motivational decline to teacher-related variables. This teacher-centric perspective persisted in Oxford and Shearin’s (1994) longitudinal analysis of 250 U. S. foreign language learners, identifying four educator-linked demotivation catalysts.

While these studies laid critical groundwork for understanding L2 demotivation, their heavy focus on external factors (e.g., pedagogical practices, classroom dynamics) resulted in a partial and learner-passive conceptualization, neglecting internal psychological and socio-cognitive dimensions (Falout and Maruyama, 2004). Zsuzsanna’s (1996) exploratory work with 15 Hungarian adolescents first identified learner-internal and contextual co-determinants, shifting focus beyond teacher-centric explanations. Building on this, Dörnyei (1998) analyzed 50 Hungarian secondary students learning English or German and proposed nine demotivation factors: (1) teacher factors, (2) undesirable teaching environment, (3) decreasing confidence, (4) negative attitude towards the target language, (5) the target language as a compulsory subject, (6) interference of anther foreign language under learning, (7) negative attitudes towards the native country associated with the target language, (8) attitudes towards peers around, and (9) textbooks and teaching materials. While Dörnyei (2001) reaffirmed teachers as a primary contributor, this multidimensional framework catalyzed later explorations of internal factors. Subsequent studies highlighted learner-internal drivers, such as negative prior language experiences (Zhou, 2012), low perceived competence (Ghadirzadeh et al., 2012), and diminished self-esteem or interest (Falout and Maruyama, 2004; Trang and Baldauf, 2007; Moiinvaziri and Razmjoo, 2014; Akay, 2017), particularly in Asian EFL contexts.

Recent studies on L2 motivation have expanded beyond traditional teacher-learner dynamics to examine systemic educational and technological factors influencing learners’ motivational trajectories. Key contextual drivers include classroom environments, assessment systems, instructional methodologies, and technology integration. For instance, Iftanti et al. (2023) observed significant motivational decline among Indonesian EFL learners during pandemic-era online learning, correlating with reduced linguistic achievement. Similarly, Zhang (2024) longitudinal analysis highlighted sustained motivation loss in hybrid learning contexts. Assessment practices have emerged as critical demotivators: Alamer et al. (2023) identified repeated low test scores as catalysts for frustration and eroded self-efficacy, while Escudero et al. (2024) documented chronic demotivation arising from persistent gaps between learner abilities and institutional expectations. Pedagogical approaches also play a role, with grammar-heavy instruction cited as a primary demotivator across diverse cultural settings (Bribesh, 2024). Additionally, overreliance on machine translation tools has been linked to learner complacency and reduced engagement, as evidenced by Klimova’s (2025) mixed-methods study, urging cautious implementation of such technologies in language education.

Existing research on L2 demotivation predominantly adopts a multifactorial framework, identifying diverse external (e.g., pedagogical practices, institutional policies) and internal (e.g., self-efficacy, affective states) factors that contribute to motivational attrition. While these studies have advanced our understanding of discrete demotivators, they largely overlook the dynamic interplay between these factors, particularly in Chinese as a Foreign Language (CFL) context. Current approaches rarely delineate how external constraints (e.g., curricular demands, sociocultural barriers) and internal psychological processes (e.g., identity conflicts, perceived incompetence) interact to shape learners’ motivational trajectories. To address this gap, this study employs Alienation Theory (Hadjar and Lupatsch, 2010; Morinaj et al., 2017), which holistically integrates environmental pressures and subjective experiences to explain disengagement mechanisms. This theoretical lens enables a systematic examination of how institutional, interpersonal, and intrapersonal factors coalesce to generate demotivation in CFL settings.

### Demotivation from an alienation theory perspective

#### Alienation theory

The concept of alienation—derived from the Latin verb alienare (“to remove” or “to separate”)—originated in theological and philosophical discourses, later expanding into sociological, historical, and educational domains (Sarfraz, 1997). Historically, it has been variably conceptualized, ranging from theological interpretations (e.g., humanity’s estrangement from divine order) to sociological critiques of modern industrial society (e.g., loss of individual agency). Seeman (1991) proposed six major variants of alienation (powerlessness, normlessness, meaninglessness, self-estrangement, social isolation, and cultural estrangement) in order to integrate various meanings of the concept.

To address conceptual ambiguities, Seeman (1991) systematized alienation into six dimensions: (1) powerlessness (perceived inability to influence one’s environment), (2) meaninglessness (absence of behavioral or belief guidance), (3) normlessness (adoption of illegitimate means to achieve goals), (4) social isolation (emotional detachment from sociocultural norms), (5) cultural estrangement (disconnection from dominant cultural values), and (6) self-estrangement (disengagement from intrinsically rewarding activities). Subsequent scholarship refined these dimensions for empirical application, particularly in educational contexts. For instance, studies on student alienation emphasize powerlessness (e.g., perceived lack of academic autonomy), social isolation (e.g., peer disconnection), and self-estrangement (e.g., disidentification with institutional goals) as key measurable constructs (Hadjar et al., 2015; Hascher and Hadjar, 2017; Hascher and Hagenauer, 2010). This multidimensional framework enables nuanced analysis of alienation as both a psychological state and a systemic phenomenon.

Empirical research has validated the multidimensional nature of alienation across diverse educational and sociocultural contexts. For instance, Khan et al. (2020) demonstrated that ESL teachers’ assessment practices—particularly the predominance of face-threatening over face-saving feedback—heightened students’ alienation during language tasks, with speaking activities showing the highest alienation levels, followed by writing and comprehension exercises. The COVID-19 pandemic further illuminated environmental drivers of alienation: Dakhi’s (2020) and Lalwani et al.’s (2023) studies linked prolonged remote learning to intensified feelings of detachment from peers and instructors, exacerbating academic disengagement. Cross-cultural investigations by Pabodha and Abeywickrama (2021) revealed that 90% of Sri Lankan students abroad experienced linguistic alienation post-arrival, manifesting as struggles in social integration, lecture comprehension, and classroom participation. Barua (2022) redefined language anxiety as a form of alienation, arguing that learners’ fear of cultural marginalization amplifies anxiety and impedes L2 acquisition. Collectively, these studies underscore alienation as a dynamic construct shaped by pedagogical, environmental, and intercultural factors.

While student alienation remains an emerging construct in educational research, its exploration has predominantly focused on Western contexts (Biasco et al., 2001). In contrast, empirical investigations of international student alienation in Chinese higher education remain scarce, despite its rising relevance amid growing transnational enrollment. Longitudinal studies reveal that initial student engagement often diminishes due to unmet academic or social expectations, leading to progressive estrangement, negative institutional attitudes, and eventual attrition (Archambault et al., 2009; Eccles and Alfeld, 2007). For instance, Moroccan students in Chinese universities may experience alienation through linguistic barriers, cultural disjuncture, social isolation, or perceived academic stagnation—factors that collectively foster a sense of marginalization within the host educational system. These observations underscore the urgency of contextualized research on alienation mechanisms in non-Western academic settings.

#### Conceptual framework

**Figure 1:**
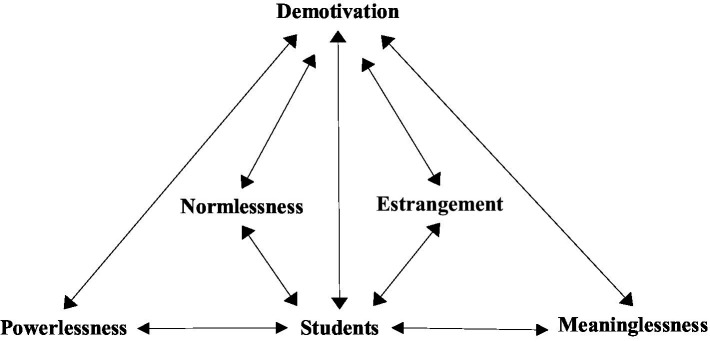
Conceptual framework.

Building on the preceding analysis, demotivation is conceptualized as a socially mediated phenomenon characterized by a perceived disconnect between learners and key educational actors or structures (e.g., peers, instructors, institutional norms), coupled with psychological distress that diminishes motivation (Dean, 1961; Galbo, 1980). Drawing from this conceptualization, Figure 1 proposes an analytical framework for L2 demotivation, grounded in its multidimensional and socially embedded nature (Rashidi et al., 2014). Rooted in alienation theory (Brown et al., 2003), the framework operationalizes demotivation through four interrelated dimensions: powerlessness (i.e., teachers), normlessness (i.e., unapproved behaviors), meaninglessness (i.e., engaged in certain or any school activities), and social isolation/estrangement (i.e., interpersonal relationship including cognitive and emotional components) as school alienation.

Demotivation in Chinese language learning emerges as a socially mediated process shaped by both systemic factors (e.g., institutional policies, pedagogical practices) and learner-related dynamics (e.g., non-conforming behaviors, perceived social isolation). The interplay of these factors may either facilitate or impede the development of learning motivation, contingent on their alignment or conflict. Grounded in alienation theory, the proposed analytical framework elucidates how demotivation arises through four interconnected mechanisms, offering a holistic lens to examine motivational attrition in Chinese language acquisition contexts.

Given the rising enrollment of international students, understanding the dual mechanisms of motivational enhancement and demotivational triggers—particularly in the context of social alienation—is critical to improving educational outcomes for both educators and learners. While existing research has explored motivational strategies, the role of alienation in shaping demotivation remains underexamined. To address this gap, the study proposes the following research questions:

1. Do Moroccan students in CFL class experience demotivation in their Chinese learning in Chinese University?
2. What alienation factors might account for the demotivation in learning Chinese among the participants?
3. Do students at different L2 demotivation levels react to alienators differently?

## Materials and methods

### Participants

Participants were 35 male and 62 female Moroccan students aged from 19 to 22 years old currently studying at a local university in Zhejiang province, PRC between March 2024 to April 2024. The enquiry took place after they had been learning Chinese for around 4 months. The questionnaire was administered to the participants during a 30-min class break. Participation was completely voluntary and anonymous.

Six participants were purposefully selected through maximum variation sampling to ensure diversity across three dimensions: academic performance (high, moderate, low), gender balance (3 males, 3 females), and disciplinary backgrounds (6 distinct majors). This strategy enabled cross-case analysis while maintaining demographic alignment with the broader study cohort (*N* = 97). The sample characteristics are shown in Table 1.

**Table 1:** Characteristics of the interviewer sample.

Name	Gender	Chinese proficiency	Background
SE	Female	HSK 1	International Economics and Trade
CH	Female	HSK 3	Business Administration
FS	Male	HSK 1	Computer Science
CB	Male	HSK 4	Chinese
NL	Female	HSK 1	Pharmacy
LL	Male	HSK 2	Mechanical Engineering

### Instrument

In this study, questionnaires and semi-structured interviews protocol were used to collect data.

We incorporated the scale of demotivation and alienation into the questionnaire design and ensured that the questionnaire was consistent with the study’s content and objectives, from item setting to question wording, and worked out “Questionnaire for Investigating Chinese Learning Demotivation among Moroccan students in Chinese University.” The questionnaire consisted of two main parts: the first part contains of the demographic information, including participant profile, concerning their gender, age and grade. The second part was the main survey, which comprises 20 items to provide an all-embracing picture of Moroccan students’ Chinese learning from the dimensions of “perceived language learning demotivation,” “learner alienation.” Questions 1 to 5 test for demotivation, and were represent with Dn. Questions 6 to 20 test for alienation, and were represent with An. The items were rated using a 5-point Likert scale ranging from 1 (strongly disagree) to 5 (strongly agree).

The measurement of perceived learning demotivation consulted Albalawi and Al-Hoorie’s (2021) demotivation model, which is a self-reported questionnaire to assess the L2 learning demotivation. The Cronbach’s alpha, of this measure, is 0.877. The measurement of learner Alienation was developed from Jessor and Jessor’s (1977) General Alienation Scale (GAS), which has been extensively used in related studies to measure learner alienation from multiple dimensions. The scale consists of 15 items, primarily measuring feelings of interpersonal alienation, uncertainty about one’s involvement in activities, and a sense of separation from others. The Cronbach’s alpha coefficient ranged from 0.80 to 0.83. All indicated good validity and reliability.

To triangulate survey findings, semi-structured interviews were conducted to collect qualitative data aligned with Brown et al.’s (2003) four-dimensional alienation framework. Each 20-min interview followed a protocol systematically addressing four alienation components: meaninglessness, powerlessness, normlessness and social isolation. Interviews were audio-recorded, transcribed verbatim, and iteratively refined through member checking to ensure accuracy. The protocol underwent linguistic validation to enhance cross-cultural clarity. For instance, the question “Does the student feel it is all right to break the law as long as he or she does not get caught?” was revised to “Some people believe it’s acceptable to break rules if there are no consequences. What is your perspective?” This adaptation preserved theoretical fidelity while improving respondent comprehension. Probing questions were dynamically adjusted based on participant responses to deepen exploration of alienation mechanisms.

### Data analysis

Data from the questionnaires were first filled into an Excel form, and then were input into SPSS 26.0 for analysis. Firstly, the data were analyzed using descriptive statistics to show the profile of the questionnaire items. Table 2 displays the descriptive statistics of participants’ questionnaire response. Both the skewness and kurtosis were between –2 and +2, enumerating that all the data is normally distributed, and they could be used for further parametric inferential analysis. It can be seen that most means of the items are between 3.00 and 4.00, except for item A2, A4, A5, A13 (concerning meaningless) barely under 3.00. More than half of the participants choose 4 (agree) or 5 (strongly agree) for item A6 and A7, which means this item perceived as more alienating by participants.

**Table 2:** Descriptive statistics for participants’ questionnaire responses (*N* = 97).

No	Min	Max	*M*	SD	Kurtosis	Skewness
D1	1	5	3.77	1.15	−0.21	−0.72
D2	1	5	3.25	1.30	−1.25	−0.10
D3	1	5	3.26	1.27	−1.13	−0.13
D4	1	5	3.22	1.18	−0.86	−0.05
D5	1	5	3.23	1.28	−0.95	−0.28
A1	1	5	3.16	1.27	−1.23	0.08
A2	1	5	2.58	1.12	−0.60	0.31
A3	1	5	3.11	1.30	−1.07	0.05
A4	1	5	2.97	1.35	−1.19	0.03
A5	1	5	2.39	1.13	−0.39	0.58
A6	1	5	3.57	1.08	−0.38	−0.43
A7	1	5	3.57	1.20	−0.50	−0.63
A8	1	5	3.32	1.26	−1.04	−0.08
A9	1	5	3.42	1.20	−0.86	−0.32
A10	1	5	3.25	1.20	−0.82	−0.12
A11	1	5	3.43	1.15	−1.02	−0.19
A12	1	5	3.19	1.28	−1.10	−0.11
A13	1	5	2.83	1.18	−0.74	0.23
A14	1	5	3.11	1.27	−1.05	−0.06
A15	1	5	3.52	1.14	−0.58	−0.47

Then, a Cronbach’s alpha reliability coefficient analysis was performed to explore the inter reliability of the questionnaire items. The reliability coefficient value is 0.895, greater than 0.8, which indicates that the reliability quality of the research data is high. After we deleted a certain number of the analyzed items, there was no significant increase in the coefficient value, indicating that most of the questions in the questionnaire should be retained. Validity was evaluated through factor analysis, with the Kaiser-Meyer-Olkin (KMO) measure confirming excellent sampling adequacy (KMO = 0.880). Bartlett’s test of sphericity was significant (χ^2^ = 689.228, df = 105, *p* < 0.001), rejecting the null hypothesis of identity correlation matrices and confirming sufficient shared variance among variables to proceed with factor extraction. These results collectively support the scale’s robust psychometric properties in measuring the intended construct (see Table 3). To answer the research questions we asked above, we performed the following steps.

**Table 3:** Reliability and validity test results of the scale.

KMO and Bartlett test		
Cronbach α		0.851
KMO		0.880
Bartlett’s test of sphericity	χ^2^	689.228
	df	105
	p	0.000

First, Pearson’s correlations were computed to determine whether significant correlations exist between the four alienators and the demotivation scores, followed by a multiple regression to examine which alienator(s) in the scale is (are) predictive of CFL performance as assessed by demotivation.

Second, a multivariate analysis of variance (MANOVA) was performed to examine alienating factor attribution differences among high-demotivating (HD), average-demotivating (AD), and low-demotivating (LD) students. The demotivation scores were reported on a scale from 5 to 25 (*M* = 16.73, SD = 4.88), according to Albalawi and Al-Hoorie’s (2021) demotivation model, which led us to divide participants into three groups based on these demotivation score thresholds (see Table 4).

**Table 4:** Academic performance level parameters.

Academic performance level	Demotivation score (Maximum: 25)	*N*
High demotivation	≥17	47
Average demotivation	9–16	47
Low demotivation	<9	3

Third, to determine how alienator(s) discourage(s) students from learning Chinese, inductive thematic analysis was used to analyze the data manually, following the procedures suggested by Braun and Clarke (2006): (1) Two researchers, FP and QYL, familiarized themselves with the data gathered through the interviews. (2) The researchers noted the initial codes that could describe the content of the collected data. (3) Codes formed were categorized into opinions based on their significance. After that, the key topics were extracted and grouped together as emergent themes. (4) The whole research team examined all the categorized extracts to determine whether they supported the themes and whether there were conflicts or the themes overlapped. (5) The final phase of report writing involves presenting evidence for each theme, ensuring the validity of the findings by illustrating extracts from participant transcriptions.

A total of 9 codes were identified and divided into four themes (Table 5). To ensure coding reliability, we implemented rigorous interrater checks throughout the analytic process. Any differences in opinions about the themes and codes were discussed together to reach a consensus. The occurrences of each participant on each of these codes were counted (present one/absent zero), and the transcripts associated with these codes were used to interpret the data. The detailed process and results are included in the Results and Discussion section.

**Table 5:** Themes, codes and number of occurrences.

Themes	Codes	*N*
Friends	No Chinese friends	6
	Close friends at home	5
HSK	Difficulty, high demands	6
	High exam fees, costly textbooks	5
	Noisy exam environment, malfunctioning equipment	2
Teachers	Humorous instruction, effective teaching, nice	6
	Strict	2
Schoolwork	Academic challenges, heavy course load	3
	Need for assistance, Software operation challenges	3

## Results

Table 6 presents descriptive statistics and zero-order correlations between the CFL university students’ alienators. As shown in the table, among the four demotivators, meaninglessness showed the highest mean, followed by normlessness.

**Table 6:** Descriptive statistics and correlations between alienators and demotivation scores (*N* = 97).

Predictor	*M*	SD	1	2	3	4	5
Demotivation	16.732	4.877	--				
Meaninglessness	31.206	6.053	0.600**	--			
Social isolation/estrangement	23.412	6.208	0.690**	0.909**	--		
Normlessness	13.309	3.658	0.650**	0.867**	0.943**	--	
Powerlessness	16.196	4.890	0.661**	0.774**	0.861**	0.819**	--

*p < 0.05, **p < 0.01.

A multiple regression was conducted to address the first research question (RQ2): Which demotivator(s) is (are) predictive of students’ Chinese performance? Results of the multiple regression are presented in Table 7. While social isolation/estrangement was the only statistically significant predictor, *β* = 0.622, *p* < 0.001.

**Table 7:** Results of regression analysis of alienators for demotivation scores.

Predictor	*B*	SEB	*Beta*
Constant	5.182*	2.055	–
Meaninglessness	−0.117	0.144	−0.145
Social isolation/estrangement	0.489*	0.231	0.622*
Normlessness	−0.024	0.298	−0.018
Powerlessness	0.252	0.145	0.252

*R*^2^ = 0.50, Adjusted *R*^2^ = 0.48, *F*(4,92) = 22.78, *p* < 0.001. *p < 0.001.

**Figure 2:**
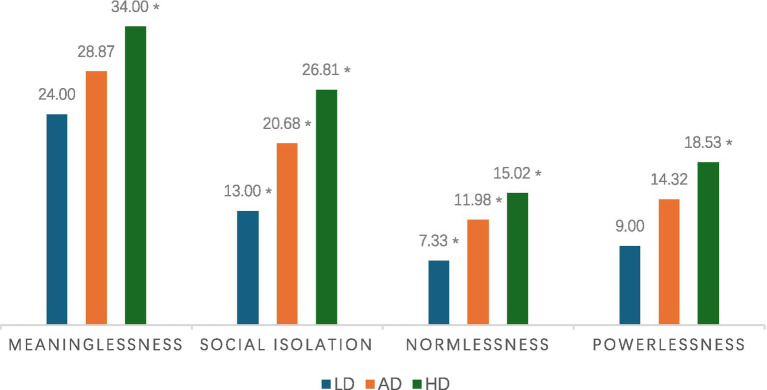
Mean alienators scores by motivation group. Note. LD = Low Demotivation; AD = Average Demotivation; HD = High Demotivation. *p < 0.05. **p < 0.001. Reference group = LD for multiple comparisons (Bonferroni).

A MANOVA was conducted to respond to RQ3: Do students at different L2 demotivation levels react to alienators differently? Pillai’s trace, an inferential test statistic for MANOVAs, revealed that students of different performance levels are influenced differently by the four alienating triggers in the CFL classroom, *V* = 0.63, *F*(5, 13) = 5.895, *p* < 0.001, Using the Bonferroni correction to control for familywise error rate (*α* = 0.05/4 = 0.0125), separate univariate ANOVAs on the outcome variables revealed a significant effect of achievement meaninglessness, *F* = 26.72, *p* < 0.001, ηp2 = 0.22, social isolation/estrangement, *F* = 23.07, *p* < 0.001, ηp2 = 0.33, and normlessness, *F* = 32.24, *p* < 0.001, ηp2 = 0.26, but a non-significant effect on teacher behavior, powerlessness, *F* = 15.80, *p* < 0.001, ηp2 = 0.25. Figure 2 summarizes the *post hoc* tests (Bonferroni) results. While the high demotivation (HD) group scored the highest across all four alienators, the attribution gaps appear to be more substantial in social isolation/estrangement, followed by normlessness.

Based on the classical theoretical framework of alienation, the four alienators were divided into two types, namely, external factors and internal factors. In our study, learners’ average ratings for these alienators endorse such a division: internal factors (i.e., meaninglessness, social isolation/estrangement) received higher ratings than external ones (i.e., powerlessness, normlessness; see Table 6). The four demotivators significantly correlate with each other at a moderate to high degree, except for the small-to-medium sized correlation between powerlessness and meaninglessness, *r* = 0.66, *p* = 0.25. Many CFL teachers strive to stimulate and maintain learners’ interest in the subject area, and our finding indicates that, to achieve this goal, only teachers’ help may not be effective as expected.

(RQ2) Which alienator(s) is (are) predictive of performance? While all four predictors in this study were positively correlated with the demotivation score, only social isolation/estrangement was found to be significant in the multiple regression model. This indicates that the semi-partial correlation between the demotivation score and meaninglessness, normlessness or powerlessness becomes trivial, after controlling for the effect of social isolation/estrangement. This finding corroborates Maslow’s (1970) theory hierarchy of needs. According to Maslow, social belongingness and love are fundamental human needs, placing them just above physiological needs like food and shelter. Social isolation threatens these needs directly, leading to feelings of alienation because humans are inherently social beings. When someone is socially isolated, they lack the essential connections, support, and sense of belonging that are crucial for psychological well-being which in turn produce motivation deficits that translate into weak performance.

(RQ3) Do students at different L2 demotivation levels react to alienators differently? Eccles et al. (2008) suggested that high-demotivators are particularly at risk of becoming alienated. This has guided us to make the high demotivation (HD) group the reference group in the *post hoc* tests following the significant MANOVA. As shown in Figure 2, the HD group suffers most across all four demotivators, but such gaps appear most substantial in social isolation/estrangement, followed by meaninglessness, and then powerlessness. No significant difference was found between average demotivators (AD) and low demotivators (LD) in powerlessness and meaninglessness. In the HD group’s ratings across four alienators, we even found that social isolation/estrangement was regarded as the most detrimental element. This finding confirmed antecedent studies conducted (Brown et al., 2003; Morinaj et al., 2017), indicating that more demotivated students tend to attribute their alienation internally (e.g., loneliness). Remarkably, average and low achievers do not differ in their demotivation attributions of meaninglessness and powerlessness, suggesting a diminished role of teachers and learning mindset in accounting for CFL performance gaps. This conclusion is contrary to most East Asia findings (Falout et al., 2009; Sakai and Kikuchi, 2009; Li and Zhou, 2013; Xie et al., 2018), which emphasize teachers’ unchallengeable authority in the classroom.

Contrary to East Asian L2 studies emphasizing pedagogical authoritarianism as a demotivation catalyst, our findings demonstrate participants’ positive affect toward instructors, suggesting that attenuated Chinese learning motivation operates independently of authoritative dynamics. This divergence may be attributed to two contextual particularities: (1) Chinese teachers’ provision of targeted emotional scaffolding for international learners, and (2) the implementation of task-based pedagogies fostering linguistic optimism. The construct of learning mindset further mediates this relationship, encompassing learners’ perceptions of education’s instrumental value across micro–macro contexts: their daily lives, familial contexts, community dynamics, or broader global issues. This belief system functions as a motivational scaffold, driving academic investment through perceived sociopersonal efficacy.

Following the conclusion drawn from our initial study, six students were interviewed to delve deeper into the findings. To present the data, the most representative transcriptions linked to each theme and code are displayed below.

In terms of “Friends,” the fact of ‘No Chinese friends’ related to social isolation were expressed by 6 participants/6. For example, we have heard “*I do not make Chinese friends*” (LL), “*I had a terrible experience with Chinese friends*” (CH), “*It’s something I’d never thought before. I did not think I could do it. But I have Chinese friends on my WeChat…but I never see them in person*” (CB), “*I know a Chinese girl. She helped me make a call to my medical insurance claim. After that, we lost touch*” (SE) or even “*We do not have Chinese in our class, so I have no Chinese friends*” (NL). Almost as many participants (5/6) expressed their longing for ‘close friends’. So, for example, we noted “*I also want to make some close friends*.” (SE), “*Making new friends is very difficult. I find it tiring to maintain a new relationship*.” (CH), “*I do not have close friends at this school. But I have several close friends next to our school*” (FS) or even “*My roommates are all from other majors, so I have to go to class alone*” (CB).

In terms of “HSK,” all participants expressed a feeling of difficult and high demanding. For example, we have heard “*We need to pass HSK 4 to graduate, but 4 is too difficult*” (SE), “*I study computer science. All of our professional courses are taught in English. It’s quite strange that we are required to pass HSK Level 4*” (NL), “*I think this requirement should be cancelled. I was initially very interested in Chinese, but the exams have made me anxious and bored*” (LL) or even “*I do not want to take those exams*.” (FS) Of these participants, five expressed the high exam fees and costly textbooks, for example: “*If you take the HSK 4, you also need to register for the KK (Chinese Proficiency Oral Test). KK is even more expensive*” (LL), “*You need to finish taking exams from Level 1 to Level 3 first before you can take the Level 4. The Level 4 exam costs 450 yuan. KK…400 yuan or 300 yuan…I cannot remember clearly. They are really expensive*.” (CH) Although HSK exams costs a lot, 2 participants complained about the bad exam environment and malfunctioning equipment, for example: “*During the exam, everyone will speak at the same time. It gets extremely noisy then. I will be affected by it*” (CB), “*During one exam, my computer did not want to work. I had to change three computers before I could log in to the exam system. I was really in a bad mood at that time*” (LL).

In terms of “Teachers,” 6 participants mentioned their teacher ‘caring and kind’. For example: “*My Chinese teacher is very gentle, and I really like her teaching way. We often carry out a lot of interesting activities*” (SE), “*During Ramadan, I did not feel well and missed several classes. My Chinese teacher did not blame me, and I’m really grateful for that*” (NL). Of these, 2 participants reported an image of strict (for example, “*Once I wasn’t feeling well and went to the hospital, but I forgot to submit the leave application. Even though I explained the situation to the teacher, I was still marked as absent*” (CH), “*The teacher of the Cultural Communication course is the strictest. Even if the leave has been approved, it will still be recorded as an absence in the attendance record*” (FS).

Regarding the “school work,” three participants spoke about their ‘heavy course load’ during the first 2 years and ‘software operation challenges’ when submit homework. For example: “*We already have a lot of professional courses, but there are even more Chinese courses*” (FS). “*Many courses require the use of Xuexitong to submit homework. But this app does not allow me to submit, always*” (CH), “*Xuexitong does not have a deadline reminder. Many times, I finish my work but cannot submit it. I have to explain the situation to the TA and ask if she can submit it to the teacher for me*” (NL).

## Discussion

### Alienation factors leading to demotivation among the participants

#### Powerlessness-mediated factors

Powerlessness, defined as learners’ perceived inability to influence their educational environment, has been widely linked to motivational attrition in L2 acquisition (Dörnyei, 2005), particularly in teacher-centric EFL contexts where pedagogical authority dominates curriculum and assessment (Li and Zhou, 2013; Xie et al., 2018). However, in Chinese as a Foreign Language (CFL) settings, while teacher-centered instruction persists, powerlessness manifests distinctively through institutional rather than interpersonal dynamics.

Analysis revealed that powerlessness exerted minimal direct influence on participants’ demotivation. A minority reported instances of overly rigid teaching methods or administrative inflexibility, which fostered perceptions of futility in seeking institutional support. For example, some students described dismissed concerns when attempting to address academic issues, exacerbating feelings of helplessness. Nevertheless, most participants perceived educators and administrators as supportive and approachable. Interviewee SE noted: “*…my Chinese teacher is so great. She really helps me a lot.”* Similarly, Interviewee CH highlighted compassionate institutional practices: *“Our Chinese class is really interesting, we have lots of good topics…the administrator is also kind, once I hurt but I forgot to ask for leave in the system, she let me submit again.*”

These findings suggest that while systemic rigidity may sporadically trigger powerlessness, positive teacher-student rapport and administrative empathy largely mitigate its demotivational effects in CFL contexts. This contrasts with EFL settings, where power imbalances more directly drive disengagement.

However, our sampled cohort, currently navigating the early stages of cultural adaptation (4 months), appears to occupy a transitional phase between the initial “honeymoon period” and subsequent acculturative challenges. As Ward et al. (2001) noted “The general satisfaction of the sojourners with their new lives, often defined in terms of their well-being.” This developmental trajectory implies potential shifts in motivational dynamics as learners progress through later adaptation phases—a hypothesis necessitating longitudinal validation via stage-specific monitoring (Galchenko and van de Vijver, 2007).

These observations partially align with Dörnyei’s (2005) motivational threshold theory while introducing a critical qualification: when institutional rigidity surpasses learners’ cultural adaptation thresholds, even robust teacher support demonstrates limited efficacy in mitigating systemic powerlessness. This threshold effect underscores the importance of proactive institutional interventions (e.g., flexible curricula, responsive administrative protocols) during early acculturation stages, which may prove more effective than retrospective support in sustaining long-term motivation.

#### Normlessness-mediated factors

Normlessness, defined as the adoption of illegitimate means to achieve socially sanctioned goals, manifests in Chinese CFL programs through institutionalized prioritization of HSK Level 4 certification as a graduation prerequisite. This policy reduces language learning to a transactional process centered on test performance, compelling pedagogical practices to align rigidly with HSK syllabi. Interview data revealed two normlessness-mediated demotivators: (1) Exam-Oriented Pedagogy: Curricula overwhelmingly focus on HSK preparation, limiting opportunities for communicative competence development. As Interviewee CB noted, **“***Learning Chinese feels meaningless—it’s just endless HSK drills*.” (2) High-Stakes Testing Pressures: Institutional emphasis on HSK pass rates incentivizes maladaptive behaviors (e.g., plagiarism, cheating), particularly when learners perceive a misalignment between test demands and their capabilities. These systemic constraints echo prior findings that credential-driven language policies erode intrinsic learning motivation (Liu et al., 2020). Most universities make HSK4 one of the graduation criteria for international students. This thus makes Chinese learning meaningless to the students but for exams.

The institutional emphasis on HSK Level 4 pass rates thus exerts systemic pressure on CFL instructors to prioritize frequent high-stakes testing aligned with exam benchmarks. This credential-focused environment incentivizes learners to adopt maladaptive strategies, including academic misconduct (e.g., plagiarism, cheating), to meet institutional demands. As Rafalides and Hoy (1971) theorized, students may rationalize norm-transgressive behaviors as necessary to fulfill externally imposed achievement thresholds, particularly when perceived capabilities misalign with test rigor. Failure to meet these expectations often results in diminished motivation for Chinese language acquisition, as learners disengage from the learning process amid perceived futility.

#### Meaninglessness-mediated factors

The analysis revealed minimal influence of meaninglessness on participants’ demotivation, likely attributable to the cohort’s strong instrumental motivation. Notably, most Moroccan students in the study self-funded their education in China, reflecting a deliberate commitment to Chinese language mastery as a pathway to academic advancement and graduate certification. This aligns with Pepanyan et al.’s (2019) assertion that international students’ determination to integrate into host societies often coexists with a pragmatic focus on credential acquisition. Central to this dynamic is learners’ perception of Chinese proficiency as a certification-driven imperative (Liu et al., 2020), wherein examinations—not linguistic engagement—serve as the primary locus of purpose. As Interviewee LL stated: “*I must pass HSK4 to graduate; there’s no alternative.*” This redefines Gardner and Lambert’s (1972) instrumental motivation framework, prioritizing institutional compliance over communicative competence, and underscores the dominance of credential pragmatism in high-stakes language learning ecologies.

#### Social isolation/estrangement-mediated factors

Social isolation, characterized by a perceived disconnect from sociocultural integration goals, emerged as a critical demotivator among participants. Interview data highlighted four interrelated factors: (1) emotional loneliness (e.g., longing for familial/friend connections), (2) lack of dependable social networks, (3) identity dissonance (e.g., tension between heritage preservation and host-culture adaptation), and (4) linguistic exclusion. As Berry’s (2005) sociocultural adaptation model posits, international students often grapple with marginalization when balancing cultural retention and assimilation. Interviewee NL’s reflection— “*I feel alone here; I miss my close friends back home*”—exemplifies this struggle, echoing Trice’s (2004) findings on the psychological toll of social detachment.

While forming bonds with culturally similar peers may alleviate isolation, such reliance can impede Chinese language immersion, as noted by Interviewee SE: “*once I hurt my leg, I need one speaking Chinese to help me make a phone call to the insurance, but I cannot…. I even feel that some locals are unfriendly to me.*” This linguistic barrier intensifies alienation for Arabic-speaking Moroccan students during crises, compounding feelings of helplessness. As noted by Pepanyan et al. (2019), domestic students may not harbor hostility towards international peers but may struggle to navigate cultural boundaries, inadvertently contributing to a sense of isolation among international students.

These findings underscore social isolation as both a cause and consequence of demotivation in high-stakes language ecologies, where linguistic and cultural marginalization erode learners’ sense of belonging and purpose.

## Conclusion

This study pioneers an alienation theory perspective on CFL demotivation, highlighting systemic and sociocultural mediators often overlooked in L2 research. Findings revealed that social isolation and normlessness emerged as the primary mediators of demotivation, driven by two context-specific factors: (1) the systemic pressure of HSK Level 4 certification as a graduation requirement, and (2) the sociocultural challenges of integrating into a localized Chinese academic environment.

The study advances current scholarship in three key ways. First, it addresses a critical gap in L2 demotivation research by applying alienation theory to understudied CFL populations, particularly Moroccan students in non-elite Chinese institutions. Second, it provides empirical validation for the utility of alienation theory in analyzing the interplay of personal, institutional, and sociocultural factors shaping motivational attrition. Third, it illuminates how credential-driven pragmatism (e.g., HSK-focused curricula) and cultural-linguistic marginalization collectively erode intrinsic learning motivation—a dynamic previously underexplored in transnational education contexts. These insights underscore the necessity of re-evaluating high-stakes language policies and fostering inclusive pedagogical practices to mitigate alienation among international CFL learners.

This study yields critical pedagogical implications for Chinese technological universities hosting international students. While educators have implemented supportive measures to enhance CFL engagement—including learner-centered instruction and culturally responsive practices—findings suggest that peer-related social dynamics, rather than teacher inadequacy, constitute the primary driver of alienation and motivational attrition. Despite participants’ widespread appreciation for instructors’ dedication (*“My Chinese teacher is incredibly supportive”*) and positive evaluations of classroom activities (*“Lessons are interactive and fun”*), many reported social exclusion and limited peer connections, which fostered resistance to the linguistic environment and diminished learning investment. To address this, institutions should prioritize structured intercultural peer interactions (e.g., mixed-nationality project teams, language partner programs) alongside curricular reforms aligning Chinese courses with students’ professional needs (e.g., discipline-specific vocabulary modules). Such initiatives would mitigate social isolation while contextualizing language acquisition within learners’ academic trajectories, thereby bridging the gap between linguistic and vocational objectives.

This study acknowledges three primary limitations. First, the exclusive focus on Moroccan students at a single Chinese university limits the ecological validity of findings, restricting generalizability to broader CFL contexts. Replicating this work across institutions and national cohorts would clarify how sociocultural and institutional variables modulate alienation dynamics. Second, reliance on self-reported data risks common-method bias, potentially inflating correlations between measured constructs. Future studies should integrate multimodal assessments—such as teacher evaluations, behavioral tracking, and institutional performance metrics—to triangulate alienation manifestations. Third, participants’ limited immersion duration (4 months) may capture transient motivational fluctuations rather than stabilized patterns. Longitudinal designs tracking learners across proficiency milestones (e.g., HSK Level 4 to 6 transitions) are needed to disentangle temporal trajectories of demotivation.

## Data Availability

The original contributions presented in the study are included in the article/supplementary material, further inquiries can be directed to the corresponding author/s.
