# Correction: Investigating the demotivation of Moroccan CFL learners from an alienation theory perspective

**DOI:** 10.3389/fpsyg.2025.1653536

**Published:** 2025-07-14

**Authors:** Fei Peng, Qiuyue Lou

**Affiliations:** College of Humanities and Foreign Languages, China Jiliang University, Hangzhou, China

**Keywords:** L2 demotivation, Moroccan CFL learners, alienation theory, mediational factors, formulating mechanism

In the published article, there was an error in the **Funding** statement. The Funding statement previously stated: The author(s) declare that financial support was received for the research and/or publication of this article. This study was funded by Zhejiang Province “14th Five-Year Plan” Higher Education Teaching Reform Project (Grant No. JGBA2024228), Research Project of the China Association of Higher Education under the Higher Education Science Research Program (Grant No. 24LX0420) and “14th Five-Year Plan” Special Project for Teacher Research and Development, China Tao Xingzhi Research Association (Grant No. ZTHJS2024131). However, the third funding source was discontinued during the project period and could no longer support the completion of the research.

The correct Funding statement appears below.

